# Machine Learning, Deep Learning, and Mathematical Models to Analyze Forecasting and Epidemiology of COVID-19: A Systematic Literature Review

**DOI:** 10.3390/ijerph19095099

**Published:** 2022-04-22

**Authors:** Farrukh Saleem, Abdullah Saad AL-Malaise AL-Ghamdi, Madini O. Alassafi, Saad Abdulla AlGhamdi

**Affiliations:** 1 Department of Information System, Faculty of Computing and Information Technology, King Abdulaziz University, Jeddah 21589, Saudi Arabia; aalmalaise@kau.edu.sa; 2 Department of Information Technology, Faculty of Computing and Information Technology, King Abdulaziz University, Jeddah 21589, Saudi Arabia; malasafi@kau.edu.sa; 3 Ministry of Health, King Abdulaziz Hospital, Jeddah 22421, Saudi Arabia; s.a.malaise@gmail.com

**Keywords:** epidemiology of COVID-19, basic reproduction rate, machine learning, deep learning

## Abstract

COVID-19 is a disease caused by SARS-CoV-2 and has been declared a worldwide pandemic by the World Health Organization due to its rapid spread. Since the first case was identified in Wuhan, China, the battle against this deadly disease started and has disrupted almost every field of life. Medical staff and laboratories are leading from the front, but researchers from various fields and governmental agencies have also proposed healthy ideas to protect each other. In this article, a Systematic Literature Review (SLR) is presented to highlight the latest developments in analyzing the COVID-19 data using machine learning and deep learning algorithms. The number of studies related to Machine Learning (ML), Deep Learning (DL), and mathematical models discussed in this research has shown a significant impact on forecasting and the spread of COVID-19. The results and discussion presented in this study are based on the PRISMA (Preferred Reporting Items for Systematic Reviews and Meta-Analyses) guidelines. Out of 218 articles selected at the first stage, 57 met the criteria and were included in the review process. The findings are therefore associated with those 57 studies, which recorded that CNN (DL) and SVM (ML) are the most used algorithms for forecasting, classification, and automatic detection. The importance of the compartmental models discussed is that the models are useful for measuring the epidemiological features of COVID-19. Current findings suggest that it will take around 1.7 to 140 days for the epidemic to double in size based on the selected studies. The 12 estimates for the basic reproduction range from 0 to 7.1. The main purpose of this research is to illustrate the use of ML, DL, and mathematical models that can be helpful for the researchers to generate valuable solutions for higher authorities and the healthcare industry to reduce the impact of this epidemic.

## 1. Introduction

The outbreak of a deadly disease called coronavirus (COVID-19) has had a significant global impact. As such, the World Health Organization (WHO) has declared it a pandemic [1]. It has affected all spheres of life; moreover, people from poor nations to developed nations are trapped indoors by the pandemic. In this situation, information and communication technologies (ICT) play an important part in connecting communities, implementing the policies, and guiding the communities by analyzing the large datasets generated from COVID-19. Within a few months after the first COVID-19 case was discovered in Wuhan, China, several researchers published articles, discussing this virus and its impact on society [2,3,4,5]. Moreover, the use of computing technologies has generated substantial support to deal with the virus. Current technological developments such as smart applications [6], Artificial Intelligence (AI) [7], Machine Learning (ML) [8], Deep Learning (DL) [9], and big data analytics [10] have led to numerous solutions, epidemiology analysis, and other clinical findings from the collected data sets. These computing technologies are also assisting healthcare and governmental agencies in controlling the spread of the virus, creating social distancing awareness, and predicting potential growth, positive cases, and mortality rates. To understand the current situation, this study mainly focused on reviewing the published papers related to ML and DL techniques. In addition, we integrated some other factors such as epidemiology, reproduction number, and virus doubling time factors in this study, which make it a different SLR than presented previously [11].

Researchers are trying to make good use of the datasets related to COVID-19 patients such as patients’ demographic data, clinical information, chest X-rays (CXR), and Computed Tomography (CT) images. For example, ML techniques assisted in preparing a learning system, and predicting the future concerns about COVID-19, using a training data set to acquire knowledge from the collected dataset [12]. It is also helpful to estimate the future trend and potential infection rate [13]. On the other side, DL implementation is providing more support by predicting the clinical findings using CXR and CT scan images [14,15]. For instance, analyzing medical images can provide irregularities in those images by highlighting different spots and predicting infected and normal patients [16]. Therefore, these computing strategies are assisting medical and governmental agencies to generate multiple findings using COVID-19 dataset, for example, severity detection, virus spreading and control, creating policies and guidelines for the communities, helping in medicine and vaccine development. 

Previously, computing scholars proposed productive health solutions to deal with different diseases and treatments [17,18,19,20,21,22]. Similarly, integration of the computing and health industries led to ideas for controlling the spread of the virus, suggestions for future virus containment, and pattern identification from real-world data. In addition, the COVID-19 pandemic has also opened many challenges that have ultimately triggered further development and integration of medical and technology fields. Whereas, ML and DL techniques helped to overcome those challenges by providing various solutions to assist the medical industry and higher authorities.

This research provides a systematic literature review and analysis of ML, DL, and mathematical models for different purposes such as predicting future cases, analyzing previous infected cases, estimating basic reproduction numbers and virus doubling time. This research discussed the number of developments and solutions provided by multiple scholars around the world. Furthermore, we discussed a number of common datasets, statistical models, and techniques to understand different factors such as infection growth rates, reproduction rates, and doubling time. The main motivation for this paper is to present a comprehensive review for the research and medical community on the current development and future challenges of ML and DL approaches for COVID-19. The summary of ML and DL techniques for prediction, detection, and treatment of COVID-19 are some of the major findings of this study. Overall, this study reviewed selected studies and contributed in the following ways:

- The main research categories can be identified in this area of study;
- Review of machine learning and deep learning techniques for understanding previous data and predicting future cases;
- Review of different mathematical models for time series analysis and estimating epidemiological factors;
- Identification of validation strategies and evaluation metrics have been used for model performance.

Accordingly, the paper is organized as follows: Section 2 discusses the methodology and search strategy applied in this study. Comprehensive analysis of ML, DL, and mathematical models applied on COVID-19 dataset is presented in Section 3. Finally, Section 4 concludes this study by highlighting future work.

## 2. Methodology and Search Strategy 

This research is mainly focused on SLR methodology. SLR is a systematic approach to organize, present, and synthesize previously published papers that can help readers to understand the current situation and potential developments in a specific field of research. Therefore, this research identified published papers that describe the COVID-19 epidemiology, use of ML and DL approaches for prediction and identification, basic reproduction rate, and virus doubling time in different regions. The subsequent sections are further describing the step-wise approach used in this article.

### 2.1. Protocol and Registration 

**Figure 1:**
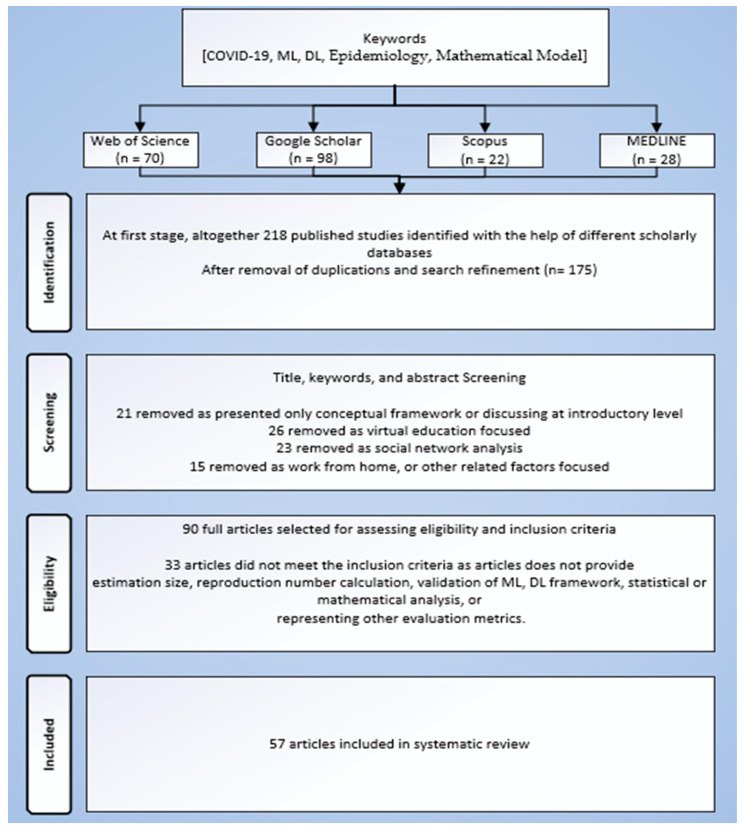
Study Selection Workflow based on PRISMA.

The systematic approach used in this study is based on the PRISMA guidelines [23]. The paper title and abstract are written as per the pre-defined guidelines. The review objectives in the introduction section were defined accordingly. The main inclusion and exclusion criteria are also discussed in Section 2.2, whereas the representation of the SLR used in this study is depicted in Figure 1.

### 2.2. Search Strategy 

We performed the searching process using different digital libraries, such as: (i) Web of Science; (ii) Scopus; (iii) Google Scholar; and (iv) Medline, up to the beginning of April 2022. This process was mainly applied under the supervision of one researcher and one clinician. Both researchers performed this task together to perform the initial screening process from computing and medical perspectives. At the first step, the following keywords were used: “COVID-19”, “novel coronavirus”, epidemiological features”, “ML or DL model prediction for COVID-19”. An enormous number of articles are available on these databases due to the large interest of researchers in this area of study. Therefore, papers were selected on the bases of explained inclusion and exclusion criteria. In the next step, the papers refined by excluding out of the scope topics, for example, social network analysis, virtual education, or work from home focused papers.

### 2.3. Inclusion and Exclusion Criteria 

We included the number of studies using specific inclusion criteria. As this research area has recorded an enormous list of publications, therefore, the inclusion criteria are important to be defined, and are also mentioned in the PRISMA guidelines document. The inclusion criteria were applied as follows: (1) the selected studies should be published in English; (2) the article must have applied and measured any of the epidemiological factors (i.e., size of estimation, epidemic doubling time, basic reproduction number, demographic features, clinical characteristics); and (3) the implementation of a ML or DL approach to identify, analyze previous cases, and predict future rate of infection and recovery. In addition, some articles were excluded due to several reasons as follows: (1) duplicate entities; (2) title, keywords, and abstract screening; (3) non-peer reviewed articles; and (4) opinion or conceptual framework focused articles.

### 2.4. Identified Research Questions 

As per the above discussion, this SLR will answer the following research questions:

- What are the main research categories that can be identified in this area of study?
- Which machine learning and deep learning techniques were proposed for predicting the future COVID-19 cases?
- Which mathematical models were used for time series analysis and for calculating different epidemiological factors?
- What validation strategies and evaluation metrics were used for measuring the model performance?

### 2.5. Quality Assessment 

Finally, the quality check process was applied by two researchers to assess the quality of the contents presented in selected studies. The main purpose of this step was to measure the quality of papers and their impact on this SLR. We used eight quality evaluation questions [24] to evaluate each article as follows: (i) objective relevance; (ii) usefulness; (iii) experimental procedure; (iv) model validation and efficiency; (v) dataset importance; (vi) availability of research limitation; (vii) discussion on future aspects; and (viii) presentation of model evaluation metrics. 

## 3. Results and Discussion 

After reviewing and analyzing the selected case studies, this section describes the major findings and discussion, as presented in different sub-sections. 

### 3.1. Characteristics of Selected Articles

The first section elaborates on the major characteristics of reviewed articles. After going through the long procedure, we short-listed 57 studies out of 218 (first search) based on their relevance to the main objectives of this study. Prior to answering the main research questions, the following are some highlights of selected articles. 

#### 3.1.1. Journal-Wise Categorization 

**Figure 2:**
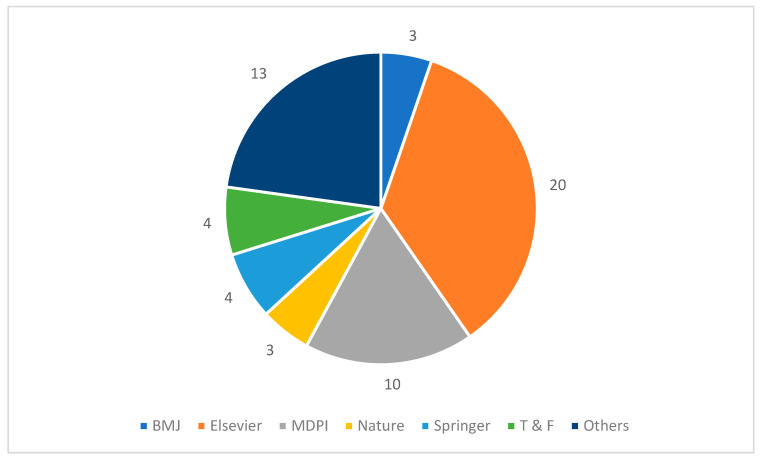
Selected Studies Publishing Journals.

Given the large number of publications in this area of research, the selection process was not basically dependent on journal venue, rather it was based on the inclusion criteria. Therefore, the researchers’ main focus was to include articles on the bases of defined rules without considering the journal venue. However, all searching databases are well-known for academic and applied research publications. Figure 2 illustrates the selected paper’s publishing venues. Most of the selected papers were published in Elsevier (20), which is one of the prominent venues for publishing quality papers. Furthermore, 10 selected articles belong to MDPI, which is one of the largest publishing venues in academic research. In the other category, we put remaining journals such as Frontiers, Wiley, IEEE, and others. 

#### 3.1.2. Country-Wise Statistics

**Figure 3:**
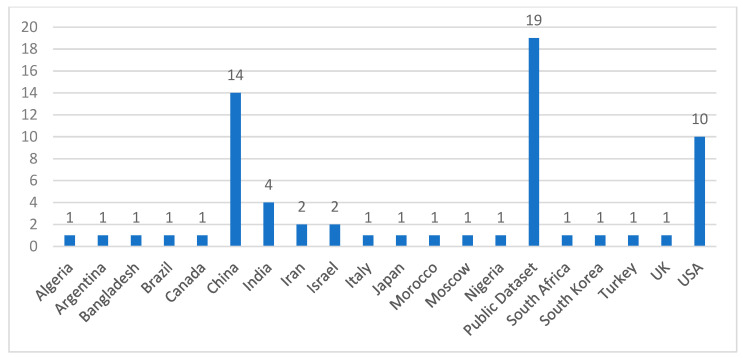
Region of Selected Studies.

We usually selected papers that proposed, implemented, and validated the prediction model using ML, DL, mathematical, or regression techniques and applied the model to the real datasets. The population of the selected case studies belonged to 19 different countries, where the COVID-19 dataset had in particular been collected and applied for different purposes, as depicted in Figure 3. Mainly, most of the studies were associated with the population of China (22%), which has been the focal point of this disease. The researchers from that region have published a number of articles related to predicting techniques [25], estimation of disease-related factors [26], and impact of prevention strategies [27]. The number of studies selected from the United States of America (USA) and the Indian regions constituted 15% and 6%, respectively. In addition, we put some studies under the public dataset category. This category represents the used dataset that either belongs to multiple regions or has been collected from an online portal (i.e., Kaggle, GitHub, and others). A large number of countries and real-world data provided a suitable ground to review the current scenario and future aspects in this area of research.

### 3.2. Research Domain 

Most of the selected studies applied prediction strategies using different kinds of models. In brief, we avoid putting most of them under the prediction category and presented them in five categories based on the main research questions mentioned in those articles. Table 1 represents the five domains classification of selected articles as follows: (i) Automated Detection; (ii) Estimation of Disease Related Factors; (iii) Impact of Quarantine and Traveling; (iv) Reporting on COVID-19 Numbers; and (v) Virus Reproduction and Doubling Time. For instance, the “Automatic Detection” category combines different prediction models implemented for automating the process of diagnosing and treatment [28]. In addition, the number of studies that belongs to this category are helpful for automatic feature extraction and improving the learning process. For the most part, those articles used CT and CXR images that played a vital role in the early diagnosis and treatment of COVID-19 disease [29].

Furthermore, the category “Estimation of Disease-Related Factors” comprises multiple studies that demonstrated other factors and their correlation with COVID-19 disease. For example, a study defined the prevalence of depression and anxiety and its associated risk factors in the patients already infected by COVID-19 [30]. High temperature & humidity [31], and geo-location [26], are some other external factors used in the selected studies to measure their impact on COVID-19 spread or control. This classification table is useful for the researchers to find a group of research papers associated with the mentioned domain.

**Table 1:** Classification of Selected Research Articles.

Research Domain Classification	Authors
Automatic Detection	[15,28,29,32,33,34,35,36,37,38,39,40,41,42,43,44]
Estimation of Disease-Related Factors	[25,26,30,31,45,46,47,48,49]
Impact of Quarantine and Traveling	[27,50,51,52,53,54]
Reporting on COVID-19 Numbers	[55,56,57,58,59,60,61,62,63,64,65,66,67,68,69]
Virus Reproduction and Doubling Time	[64,65,66,70,71,72,73,74,75,76,77,78]

**Figure 4:**
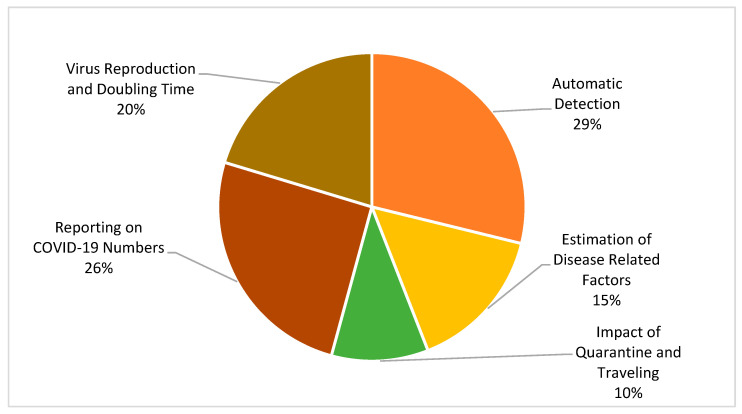
Research Domain Classification.

Predicting COVID-19 is handled in different ways and perspectives, from its detection to prevention there are so many areas where researchers have proposed computing solutions. The categories shown in Figure 4 portray the percentage of selected articles in different domains. “Virus Reproduction and Doubling Time” is the third largest category in this SLR and comprises 20% of the 57 articles. These articles reported epidemic doubling time and basic reproduction rate using previous data [73]. Overall, these estimates were useful for governmental authorities to prepare a number of guidelines for breaking the chain of COVID-19 infection. 

### 3.3. Types of Modeling Applied for Modeling COVID-19 Cases

**Figure 5:**
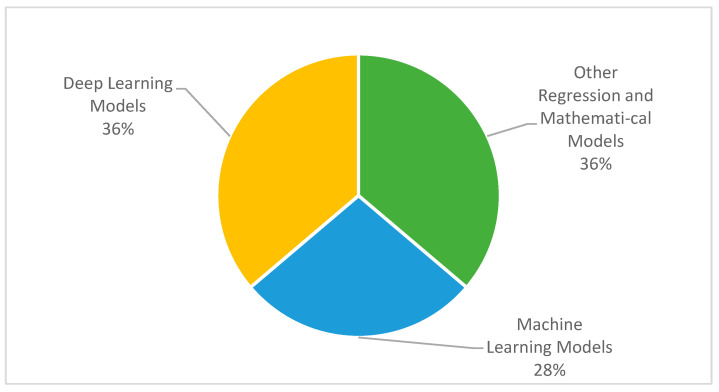
Types of Modeling in Selected Studies.

The number of research domains discussed above has applied ML, DL, mathematical, or regression models. For the medical image classification task, DL techniques are considered feasible and suitable for automatic feature extraction and finding out the hidden patterns from those images. On the other side, a large number of ML algorithms are applied for the classification, identification, and analyze of COVID-19 cases. Figure 5 represents that 28% of the selected papers applied ML techniques, whereas 36% implemented DL, or other mathematical models, respectively. 

The mapping of each article with modeling techniques is shown in Table 2. It can be evident from this table that all kinds of models are almost equally important and proposed several solutions while dealing with COVID-19. It summarizes that 21 out of the 57 selected articles used DL approaches, 16 out of the 57 employed ML, and a final 21 articles used other regression or mathematical models. Whilst the regression model is one of the ML techniques, we put regression models in the “Others” category, due to their dynamics, variety, and association with mathematical and statistical approaches. A detailed review of each type of modeling is presented in the subsequent sections. 

#### 3.3.1. Machine Learning Models

**Figure 6:**
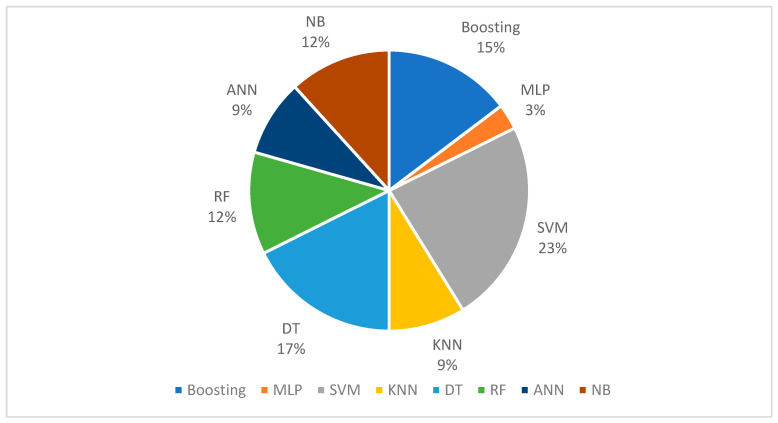
Ratio of ML Models in Selected Studies.

Of the selected studies, 28% of the studies implemented ML techniques to propose learning procedures or to develop prediction models. As shown in Figure 6, over 23% of the articles employed support vector machines (SVM), whereas 17% Decision Trees (DT), 15% Boosting, 12% Naïve Bayes (NB) and Random Forest (RF), 9% Artificial Neural Net (ANN) and K-Nearest Neighbor (KNN), and MLP implemented recorded the lowest %, at 3%. Previous studies highlighted the importance of the ML algorithm for multi-purpose solution building, which was further justified through measured accuracy of the models. For instance, research was applied to the multi-region datasets for (i) predicting the spread of virus in different regions; (ii) virus transmission rate; (iii) ending point; (iv) weather conditions and their association with the virus [55]. 

Early assessment and identification of COVID-19 is helpful for effective treatment and it can also reduce the healthcare cost. A study used multi-ML models for predicting the infection status in different states of India [67]. Overall, 5004 patients were recorded with a cross-validation approach used for model implementation. For this, the ensemble model proposed using different classifiers such as SVM, DT, and NB. The model outperformed (accuracy: 0.94) as compared to other studies 0.85 [79] and 0.91 [80]. 

The use of ML approaches for COVID-19 disease recorded several frameworks. One study analyzed the multiple symptoms to identify risk factors for clinical evaluation of COVID-19 patients [49]. The study used 166 patients of different age groups including demographic features, disease history, and other test information. The study applied a multi-model (ANN, SVM, and Boosting) approach, in which ANN outperformed other classifiers with 96% accuracy. Moreover, it is also useful for real-time forecasting purposes, as discussed in a study applied to the time series data collected from Johns Hopkins [56]. The model provided predictions for the next 3 weeks and the results were suitable for the higher authorities to plan resources and prepare policy accordingly. In the same way, another study proposed a model using SVM and DT that forecasted the next six months in Algeria [57]. 

Scholars suggested ideas to support the government by predicting numbers on potential virus growth using different variables. In the study, factors such as weather, temperature, pollution, gross domestic product, and population density were used to develop a prediction model [25]. The collected dataset was associated with the different states of the USA. SVM, DT, and regression-based models were applied in this study to forecast the spread of the virus. SVM performance showed 95% more variation than other models. The study further suggested that population density can be a critical factor to analyze the size of the spread. The author explored a good factor, but comparing this factor in high and low population regions can provide better results. In addition, the impact of quarantine was measured using data collected from three countries (Italy, South Korea, and the USA) [51]. The study recommended that strict government policies for isolation played a significant role in halting the virus’ spread. 

The review process in this study identified several facts about ML techniques. According to the studies selected in this paper, the most useful model is SVM, which has been used in 23% of articles. DT (17%) and Boosting (15%) stand in second and third place. Based on the review performed on selected case studies, the ML approach is useful to predict future growth [55], severity detection [47], analyzing CT radiomic features [63], CT images’ classification [37], measuring the impact of social restrictions on virus spread [27], the importance of travel restrictions in reducing virus spread [52], measuring depression and anxiety in COVID-19 infected people [30], and using population density as the main factor for prediction [25]. The model evaluation has shown extraordinary performance in different studies, such as for severity detection (Classifier: SVM, Accuracy: 81%, China) [47], CT images classification accuracy (Classifier: SVM, Accuracy: 99.68%, China) [37] (Classifier SVM, Accuracy: 92.1%, Multi-region) [55], and spatial visualization (Boosting, R^2^: 0.72, China) [26]. 

#### 3.3.2. Deep Learning Models

**Figure 7:**
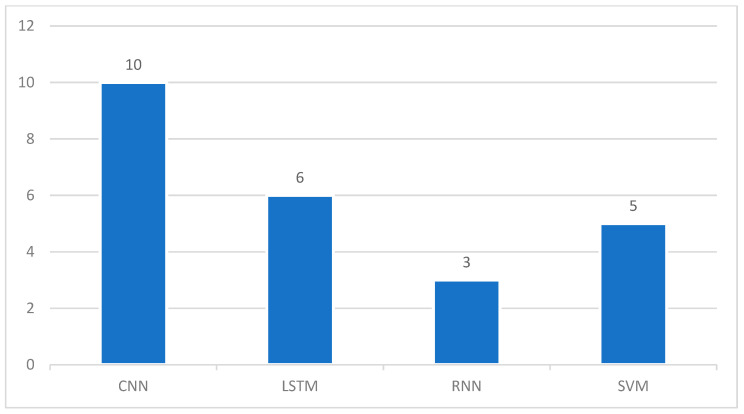
Ratio of DL Models in Selected Studies.

Another major development presented in this SLR study is to review published papers that performed DL techniques to automate the COVID-19 detection process and predict a number of cases. Fast diagnostic methods and deep analysis can help and control COVID-19 spread and that is strongly supported by DL methods. In this SLR, based on the review performed on the selected cases, Figure 7 elaborates on the DL models and the number of times they are used in selected studies. The figure explains the usefulness of the Convolutional Neural Network (CNN) model as it has been used in 10 different articles from the selected studies. Although LSTM is the modified version of Recurrent Neural Network (RNN), to be more specific, we kept them separated and used the same name as mentioned in the studies. Altogether LSTM and RNN were used in nine different articles.

The use of a CNN-based deep neural system for medical image classification has been known for its better feature extractions’ capabilities [15,29]. A research team proposed and used 10 different types of CNN-based models to classify the images into infected and non-infected groups [32]. For this, 1020 CT images, and 108 patients’ records were used for the model implementation and validation process. ResNet-101 and Xception showed the best performance with accuracy measured as 99.51% and 99.02%, respectively, although high accuracy could be tested by adding more images from different classes. In addition, research applied the CNN technique to distinguish the infected and non-infected person using their CXR images. For better accuracy and automatic feature detection, transfer learning with CNN approach applied which helped to achieve accuracy, sensitivity, and specificity as 96.78%, 98.66%, and 96.46%, respectively [28]. 

As per the recommendations collected from different studies, DL approaches could be helpful in several situations. Commonly, different studies used CNN methods to classify CT and CXR images (Classes: COVID-19 infected, viral pneumonia patients, normal patients) [34,36,44], whereas model accuracy recorded more than 90%. In addition, these strategies most of the time used a split validation approach. Another study proposed CNN-based architecture (STM-RENet) to analyze and identify radiographic patterns and textural variations in CXR images of COVID-19 infected people [39]. The proposed model achieved an accuracy of 96.53%, which can be adapted for detecting COVID-19 infected patients. COVID-Net, a CNN-based network system for automation in clinical decisions [35], detection of COVID-19 using SVM classifiers [42], and predicting severe and critical cases based on clinical data of patients using SVM classifiers [33] are some other valuable researches that can provide potential feedback to the medical and higher authorities. 

The idea of providing a more robust forecast is presented in a research paper with the help of the LSTM framework and mathematical epidemic model [64]. The paper proposed a model that can predict the number of cases on daily bases for the next 15 days with reasonable interpretation. Similarly, another integration was presented using LSTM and Auto-Regressive Integrated Moving Average (ARIMA) techniques, that can forecast for the next 60 days [65]. LSTM has been applied in another study that used time series analysis, evaluated the model, and forecast the number of cases for the next 15 days, applied to the Moscow dataset [59]. 

The implementation of DL models assisted positively in this epidemic situation to encounter the issues related to automatic infection detection using CT or CXR [43], finding out hidden features [48], forecasting for the next few days [68], and correlating external factors with COVID-19-like social restrictions [27], or spatiotemporal data [50]. According to the selected studies, the range of forecasting provided was from 15 to 60 days. The most common evaluation metrics used were RMSE and MAPE. In addition, for classification tasks the common evaluation metrics used were sensitivity, specificity, and accuracy, which most of the time measured more than 90% [38,41]. 

#### 3.3.3. Others (Regression and Mathematical Models)

**Figure 8:**
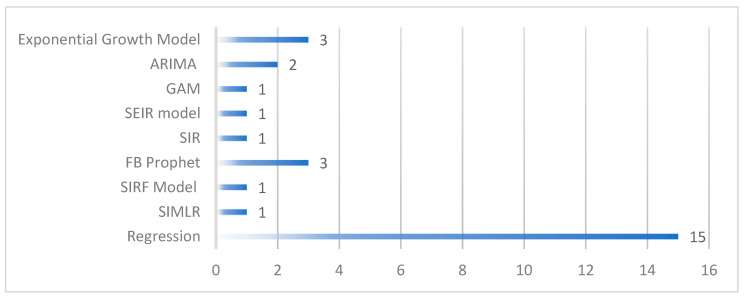
Ratio of Mathematical and Regression Models in Selected Studies.

This category combines different mathematical, statistical, regression, and compartmental models that provided a number of solutions in this epidemic situation. These compartmental models use groups of populations and employ mathematical equations using different disease-related factors [24]. These models are also helpful for early prediction, growth rate, number of deaths, and recoveries, which ultimately can provide assistance to higher authorities in controlling the situation. Figure 8 represents the number of models covered in this category and used in selected case studies. Regression analysis (15) is at the top, which has been proved several times to apply time series analysis and forecast for future infections. In addition, the exponential growth model, the SIR Model (Susceptible, Infectious, Recovered), and its extended version such as SEIR (Susceptible, Exposed, Infectious, Recovered), SIRF (Susceptible, Infectious, Recovered, Fatalities), and SIMLR (Susceptible, Infected, Machine Learning, Recovered) are used in selected cases.

SIMLR is an extension of the basic epidemiological SIR model that is integrated with the ML approach, applied to track the changes in policies and guidelines applied by governmental authorities [58]. The main purpose of this model was to forecast one to four weeks in advance in Canada and the United States. The results generated and presented a comparison of MAPE in different states. Using a dataset up to July 6, 2021 (India and Israel) the SIRF model was proposed, which extended the basic SIR model by adding fatalities data and can forecast for the next 100 days [60]. In addition, the third extended version found in the selected studies is SEIR, integrating with the “exposed” parameter. This study proposed a simulation-based approach applied to the past 300 days’ data from China to see the impact of prevention strategies [53].

Multiple regression models were applied in a study to predict the number of positive cases in the next few days [25,30]. The idea was to strengthen government policies in order to reduce the number of infected people [66]. For forecasting purposes, the study collected data (22 January 2020, to 12 July 2021), where the study suggested that if the current number of cases are 5000, it can be doubled in the next 5 days. Similarly, the linear regression method was applied to estimate the basic reproduction rate based on the data (1 March–18 May 2020) collected from different regions of the United States [46]. The main idea of this study was to analyze the impact of face-coverings in different states. The result estimated that the total number of infections at the end of May could reach up to 252,000, which shows the positive impact of face coverings. The regression model was applied in different studies and highlighted multiple factors, such as higher temperature, which would help to reduce the transmission rate in China and the USA [31], while the study conducted in Brazil did not support the same idea [45]. Some other time series forecasting models such as FB Prophet applied in Bangladesh (estimation size: 8 March 2020 to 14 October 2021) [61], India and Israel (estimation size: July 6, 2021) [60], ARIMA in China (estimation size: 22 January 2020 to 7 April 2020) [69] are some useful models that can help their country’s representatives to prepare guidelines and prevention strategies. 

#### 3.3.4. Model Validation Strategy

**Figure 9:**
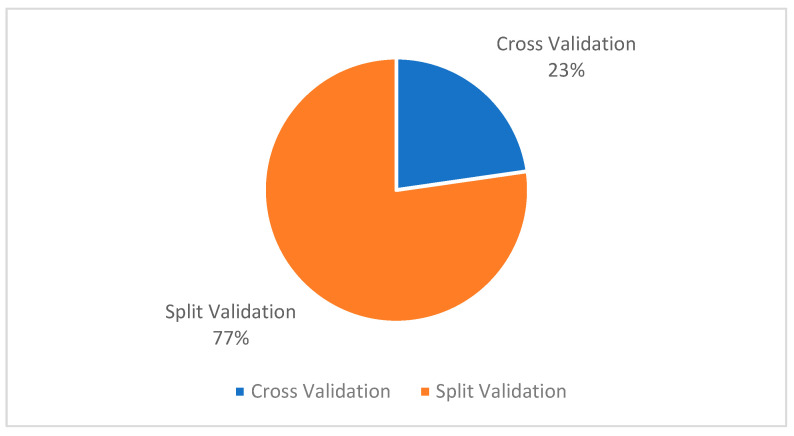
Ratio of Validation Strategies in Selected Studies.

In this section, we elaborate on the number of validation strategies applied in selected case studies and their ratio, to understand the most favorable validation method in the current situation. As shown in Figure 9, most of the selected studies employed split validation (77%) strategies. One of the reasons behind split validation could be the availability of a smaller number of datasets. As per the importance and quality of cross-validation strategy discussed in previous studies [81], it could be a critical point for the future researchers to: (i) encourage dataset availability on the public platforms; (ii) assess the difference between both validation strategies. 

#### 3.3.5. Quality Evaluation Metrics Used in Selected Studies 

**Figure 10:**
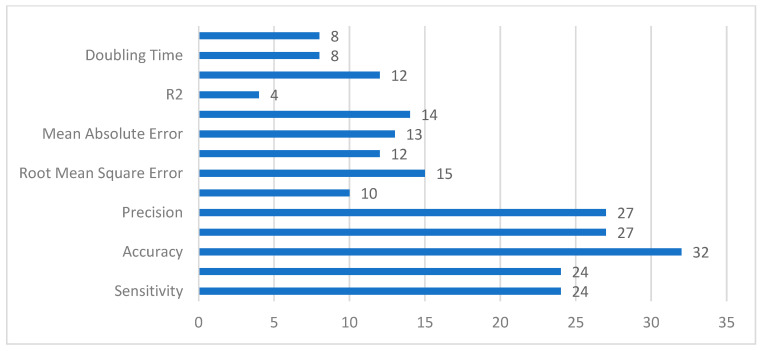
List of Evaluation Metrics used in the Selected Studies.

The evaluation metrics allowed researchers to quantify the work presented in any study. It also allowed the author to present the results in an efficient manner. However, the selection of the evaluation metrics is an important aspect, which is based on the type of model employed in that study. The list of quality metrics used for model evaluation in selected studies is depicted in Figure 10. The important thing to mention here, these numbers are not representing the best or worst evaluation metric, they are just presented to highlight the number of potential metrics that could be used, based on the type of forecasting model. Commonly, after reviewing all papers, we can say that growth rate, doubling time, *R*_0_, R^2^, MAPE, MAE, MSE, and RMSE are evaluation metrics that are useful (but not limited to) for time series, regression, compartmental models, or for other mathematical models. The remaining are possible evaluation metrics when we employed other ML or DL methods. 

### 3.4. Epidemiologic Characteristics and Transmission Factors

This section describes epidemiological and transmission factors reviewed from the selected case studies. We present the major findings in two sub-sections: (i) Epidemic Doubling Time; and (ii) Basic Reproduction number as presented in subsequent sections.

#### 3.4.1. Estimated Period and Doubling Time

The epidemic’s exponential growth within a short period is reported from all over the world. Different studies proposed solutions to reduce, control, and mitigate the impact of COVID-19. The main purpose of those studies was to provide some useful numbers to the higher authorities for preparing controlling strategies as illustrated in Table 3. Therefore, research conducted in India using the data collected from February 2020 to March 2021, estimated that the epidemic doubled in size every 1.7 to 46.2 days. The minimum and maximum numbers were calculated based on the infected cases in different districts [70]. Using linear regression and SVM approaches, an analysis was conducted on multi-region data, where the mathematical model estimated the size on the basis that if the number of positive cases is 5000, it will double in size every 5 days, whereas 163,840,000 cases would be doubled in 140 days. The equation presented multiple scenarios using different datasets, to make the government aware about the severity level of the epidemic [66].

Using a similar strategy (the exponential growth model) estimated the doubling size in China was every 3.6 days [75], whereas another Chinese study concluded that the doubling size was every 4.2 days [74]. The number of studies presented and reviewed in this study conducted in different regions highlighted multiple factors for the governmental agencies. According to the selected cases, the interval for doubling time occurs between 1.7 to 140 days, based on the number of infected people and estimation size. The recommendations list collected from different articles are compiled and presented in the following table. 

#### 3.4.2. Basic Reproduction Number (*R*_0_)

Basic reproduction number estimation plays a significant role and directly impacts different factors such as procedures, guidelines, travel restrictions, quarantine process, and other related factors. Table 4 represents the *R*_0,_ identified in selected case studies. Generally, a larger reproduction rate would have a large number of infected people in the future. Mainly, the exponential growth model, SIR, ARIMA, and other mathematical models are used for measuring the rate of reproduction number. In addition, the interval of ranges based on the given studies occurs between 0 to 7.1. In which, 0 is the ideal case discussed in the paper related to some districts in India, which recorded less than 40 isolated cases and no local transmission of infection reported [70]. 

The highest *R*_0_ estimate of 7.1 was measured for the New Jersey, USA, in a study published recently [72], which indicates the virus transmission varies in different states. Another recent study used SIR and applied it to the dataset collected from Spain with ranges for *R*_0_ from 0.48 to 5.89. As mentioned in the study, the minimum value is clearly identifying the impact of lockdown as the *R*_0_ dropped from 5.89 (before lockdown) to 0.48 (after lockdown) [73]. 

## 4. Conclusions

As we are aware, the pandemic has had an impact on the entire world. This research discussed the role of ML and DL techniques that can assist medical and governmental agencies. This SLR reviewed a number of papers to identify ML, DL, and mathematical models that can predict the potential impact, transmission growth rate, and virus identification. The research identifies that understanding epidemiology and forecasting models are important to mitigate the impact of this epidemic situation. As for now, the virus transmission is continuing to spread around the world, and the integration of multiple strategies can help to control the situation. In the future, we need to select the most recent papers, while presenting the work using different SLR tools. We discussed a number of key findings that can be helpful for policymakers and future researchers. This type of study should be conducted in the future to understand, analyze, and collect the recent advancement in this area of research.

## Figures and Tables

**Table 2:** Number of Articles and Types of Modeling in Selected Studies.

Types of Modeling	Authors
Deep Learning Models	[15,27,28,29,32,33,34,35,36,38,39,41,42,43,44,48,50,59,64,65,68]
Machine Learning Models	[25,26,27,30,37,47,49,51,52,54,55,56,57,63,67,72]
Others (Regression and Mathematical Models)	[25,30,31,45,46,53,58,60,61,62,64,66,69,70,71,73,74,75,76,77,78]

**Table 3:** Epidemic Doubling Time in Selected Studies.

Author	Country	Method	Dataset	Doubling Time	Tool Used	Recommendation by Author
[70]	India	Exponential Growth Model	February 2020–March 2021	1.7 to 46.2 days (based on districts)	Q-GIS software	no uniformity across country to analyze and study epidemics in future
[74]	China	Global Epidemic and Mobility Model (GLEAM)	By 23 January 2020	4.2 days	-	travel restrictions
[66]	Multi-Countries	Linear Regression and Support Vector Machine	22 January 2020, to 12 July 2021	Min = if (5000 cases) double in 5 days Max = if (163,840,000 cases) double in 140 days	-	government and individuals aware about the severity
[75]	China	Exponential Growth Model	1–23 January 2020	3.6 days	-	prevention measures were effective
[76]	South Africa	Susceptible–Exposed– Infectious–Recovered (SEIR) model	By 23 November 2021	3.3 days	-	immune evasion is more concerning increased transmissibility
[78]	Argentina	Agent-based Model	Multiple Scenario	2.0 to 7.14 days		social distancing measures

**Table 4:** Epidemic Basic Reproduction Number in Selected Studies.

Author	Country	Dataset	Basic Reproduction Number	Method	Confidence Interval (CI)	Tool Used
[71]	China	1–15 January 2020	2.56	Exponential Growth Model	95% CI	-
[70]	India	February 2020–March 2021	0 to > 7 (based on district)	Exponential Growth Model	-	Q-GIS software
[72]	USA	21 January 2020–21 June 2020	2.3 to 7.1 (based on different states)	Bayesian inference	95% CI	PyBioNetFit
[73]	Spain	March–April 2020	0.48 to 5.89 (different conditions)	SIR (Susceptible-Infected-Recovered)	95% CI	-
[64]	USA	22 January 2020–10 August 2020	2.747 to 3.856 (increase as days increase)	Mathematical Epidemic Model (MEM) + DL	-	MATLAB
[65]	Morocco	22 January 2020–22 November 2020	0.9 and 1.3 (increase as days increase)	Auto-Regressive Integrated Moving Average (ARIMA) and Long short-term memory (LSTM)	95% CI	Python
[74]	China	By 23 January 2020	2.57	Global Epidemic and Mobility Model (GLEAM)	90% CI	-
[75]	China	1–23 January 2020	4.2	Exponential Growth Model	95% CI	-
[77]	Malaysia	1 February 2020–8 November 2020	3.96	Susceptible-Exposed-Infectious-Removed (SEIR) Model	95% CI	Excel
[31]	China, USA	By 10 February 2020	0.023 (China) 0.020 (USA)	Retrospective Regression Analysis	95% CI	Python
[46]	USA	8 March–12 April	3.96	Linear Regression	95% CI	-
[25]	USA	By 16 April 2020	3.81 to 4.07 (based on method)	SIR (Susceptible-Infected-Recovered)	95% CI	-

## Data Availability

Not applicable.
